# Corn Cobs’ Biochar as Green Host of Salt Hydrates for Enhancing the Water Sorption Kinetics in Thermochemical Heat Storage Systems

**DOI:** 10.3390/molecules28145381

**Published:** 2023-07-13

**Authors:** Minh Hoang Nguyen, Mohamed Zbair, Patrick Dutournié, Lionel Limousy, Simona Bennici

**Affiliations:** 1 Université de Haute-Alsace, CNRS, IS2M UMR 7361, F-68100 Mulhouse, France; minh-hoang.nguyen@uha.fr (M.H.N.); mohamed.zbair@uha.fr (M.Z.); patrick.dutournie@uha.fr (P.D.); lionel.limousy@uha.fr (L.L.); 2 Université de Strasbourg, F-67000 Strasbourg, France

**Keywords:** thermochemical energy storage, solid/gas sorption, biochar, salt hydrate, material characterization

## Abstract

Heat storage technologies are essential for increasing the use of solar energy in the household sector. Their development can be achieved by designing new storage materials; one way is to impregnate a porous matrix with hygroscopic salts. In this article, the possibility of using biochar-based composite sorbents to develop promising new heat storage materials for efficient thermal storage is explored. Biochar-based composites with defined salt loadings (5, 10, 15, and 20%) were produced by impregnating MgSO_4_ into a biochar matrix derived from corn cobs. The new materials demonstrated a high water sorption capacity of 0.24 g/g (20MgCC). After six successive charging-discharging cycles (dehydration/dehydration cycles), only a negligible variation of the heat released and the water uptake was measured, confirming the absence of deactivation of 20MgCC upon cycling. The new 20MgCC composite showed an energy storage density of 635 J/g (Tads = 30 °C and RH = 60%), higher than that of other composites containing a similar amount of hydrate salt. The macroporous nature of this biochar increases the available surface for salt deposition. During the hydration step, the water molecules effectively diffuse through a homogeneous layer of salt, as described by the intra-particle model applied in this work. The new efficient biochar-based composites open a low-carbon path for the production of sustainable thermal energy storage materials and applications.

## 1. Introduction

Thermochemical heat storage (TCHS) systems that are very broadly implanted in the areas of solar photo/thermal utilization, building insulation, and industrial applications are becoming increasingly important as a means to increase the energy efficiency [1]. TCHS systems have an important industrial application value to guarantee the adaptability and stability in renewable energy implementation. TCHS systems increase the utilization efficiency as a result of their high heat storage capacity, the possibility of achieving long-lasting energy storage period, and the negligible energy loss during storage [2]. Salt hydrate-based TCHS materials have emerged as the best candidate for storage materials due to the simple reaction involved (hydration), the non-toxicity, and the absence of side reactions [3,4]. Most TCHS operates on the principle of the reversible sorption reaction (generally water sorption), where heat is stored during desorption (charge) and released during adsorption at a later time (discharge). To convert the system into a practical residential application (space heating and domestic hot water generation), the TCHS material must be carefully selected. The selection criteria for salt hydrates [5,6,7] include a high energy density, a low charging temperature with a good mass and heat transfer [8], and an improved thermal conductivity [9]. The working pair MgSO_4_–H_2_O has gained attention [8,9] due to the high theoretical energy density (2.8 GJ/m^3^) and the high deliquescence relative humidity (DRH) of 90%. However, overhydration, aggregate formation during rehydration, kinetic hindrance limiting mass and heat transfer, and poor cyclability [10] are the major drawbacks of salt hydrate-based TCHS. Consequently, the potential of the system has not yet been achieved, and due to these constraints, the energy storage capacity is still too low. Attempting to make composites by dispersing the salt in a porous matrix to prevent swelling and aggregation of the salt is one way to take advantage of MgSO_4_’s great potential and overcome one of the main materials’ drawbacks: mass and heat transfer limitations. To achieve the goal of efficient and low-temperature thermochemical heat storage, it is necessary to design and synthesize novel TCHS composites that not only present excellent storage capacity and hydration behavior, but also assure a long hydration/dehydration cycle reliability. The goal can be reached by finding suitable support for dispersing the salt hydrate to minimize the thickness of the salt deposit and consequently improve the water molecule diffusion. A promising candidate is biochar.

In contrast to its widespread application in other domains such as conversion and storage of energy [11], supercapacitors, and batteries [12,13], to the best of our knowledge, there is no study reporting on the application of biochar materials derived from biomass waste in the field of thermochemical heat storage by sorption.

Biomass is predominantly composed of biopolymers such as cellulose, hemicellulose, and lignin [14], which serve as a suitable carbon skeleton framework for the production of carbonaceous materials such as biochar. Bamboo bagasse [15], cotton rosewood [16], and pinewood [17] are some examples. A diverse spectrum of biomass precursors rich in biomolecules such as carbohydrates and proteins contributes to significant scientific progress in the development of functional biomass-based carbons [18]. A particularly renewable and sustainable source of biochar is woody biomass, such as agriculture’s waste [19,20].

Pyrolysis is a sustainable method of converting biomass into biochar and biofuels. It consists of the thermal conversion of biomass in an oxygen-free or oxygen-poor conditions [21,22]. Due to its distinctive physicochemical characteristics, the biochar has a wide range of potential uses in a variety of fields, including energy production, soil conditioning, soil remediation, and catalysis [23,24,25,26,27]. The pyrolysis temperature is the most important property of the resulting biochar [28,29]. Biochar produced at high pyrolysis temperatures (>600 °C) has a high pH, a large surface area (porosity), and a high aromaticity. Lower process temperatures, on the other hand, result in higher char yield, a higher amount of volatiles (that recondense on the biochar surface), and oxygen-containing surface functions that provide higher electrical conductivity and cation-exchange capacity [25,30,31]. 

Moreover, it should be mentioned that biomass is an entirely renewable energy source because the CO_2_ generated during its combustion and utilization processes does not account for the contribution to the atmospheric CO_2_ (because of the neutral carbon balance due to its biogenic origin). In other words, plants utilize CO_2_, which is released into the environment as a result of other plants’ decomposition processes for growth and metabolic functions [32]. Therefore, using biomass accelerates the release of CO_2_ into the atmosphere but does not affect the global carbon balance; once released, CO_2_ will be metabolized by plants to create new biomass [33]. 

Biochar derived from biomass waste has already displayed excellency in energy storage and catalysis due to its valuable advantages of a low carbon footprint [34], wide availability, and low cost [35,36]. Moreover, the annual production of biomass is around 130 billion tons worldwide [37]; this massive quantity assures the possibility of producing biochar in sufficient quantities for the eventual development of the TCHS sector. Converting the biomass waste into a support for producing TCHS materials is an interesting idea: such composites can store and convert renewable energy through a sustainable technology, thus applying a sustainable material and contributing to sustainable development [38]. 

In this study, MgSO_4_ was incorporated into a porous biochar support derived from corncobs pyrolysis. The corresponding MgSO_4_ TCHS composites were synthesized, carefully characterized, and examined to develop adsorbent materials that successfully disperse the salt over the support surface. Additionally, to gain a better understanding of the effect of MgSO_4_ salt, studies on the hydration and energy release behavior of the materials have been detailed.

## 2. Results and Discussions

### 2.1. Composite Adsorbents’ Structural and Textural Characteristics

**Figure 1:**
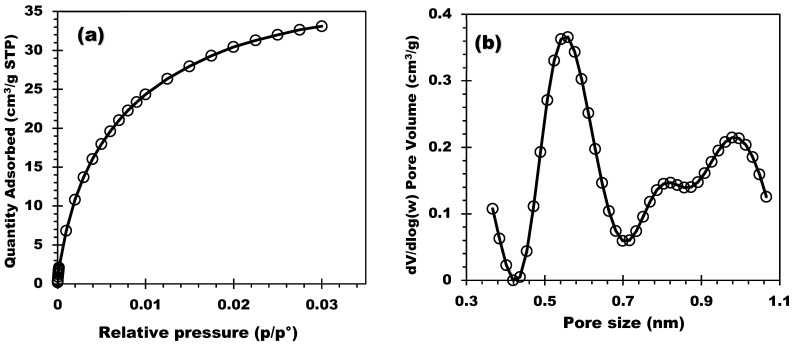
(**a**) CO_2_ adsorption isotherm and (**b**) Pore size distribution of CC550.

The chemical composition determined by WDXRF (2nd column) is outlined in Table 1. The support was characterized by CO_2_ manometric adsorption, and the isotherms were collected at 0 °C. Figure 1a,b display the CO_2_ adsorption isotherm and the pore size distribution (PSD) of the biochar, respectively. PSD was obtained by applying the CO_2_–DFT model. The PSD clearly showed the microporous structure of the biochar and, in addition, the existence of ultramicroporosity (conventional for biomass-derived carbon materials). Three populations of pore size were detected: the first centered at 0.56 nm with a sharp and clear peak, and two others centered at 0.82 and 1.00 nm. The specific surface area S_BET_ and the pore volume V_p_ were then determined, and the result showed S_BET_ = 175 m^2^/g and V_p_ = 0.06 cm^3^/g due to the ultra-micro and microporosity. 

**Figure 2:**
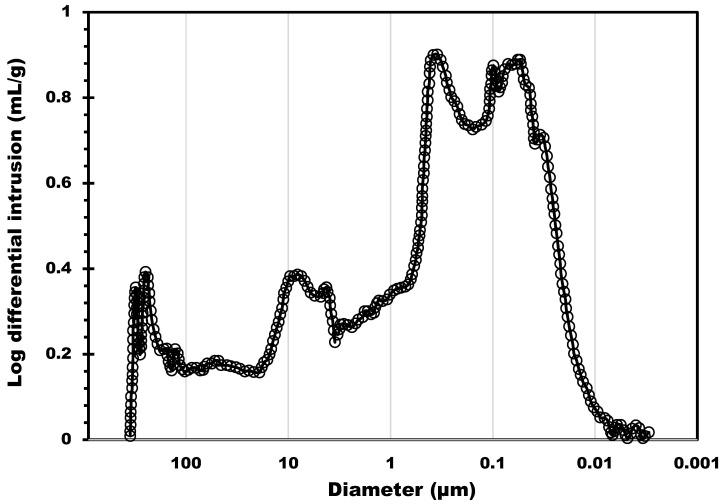
Pore size distribution determined by Hg porosimetry analysis of the biochar support.

**Figure 3:**
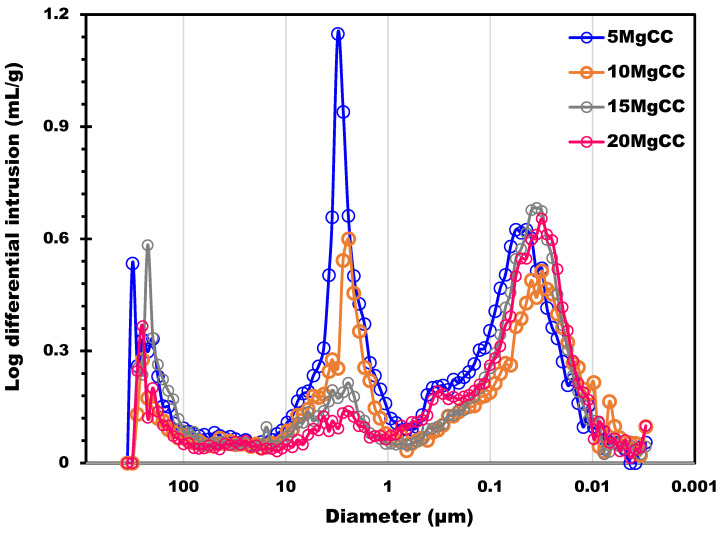
Pore size distribution determined by Hg porosimetry analysis of the four composites.

The biochar support was also characterized by mercury intrusion porosimetry to probe the macroporosity. The estimated total pore area due to macrospores was 78.4 m^2^/g (this surface was estimated considering perfectly cylindrical pores). Figure 2 shows the distribution of the macropores size from 300 µm to about 0.01 µm; three main pore size ranges were identified. The first was centered at 8 µm (in the 20 to 3 µm range), the second at 0.37 µm (characterized by a band in the 0.7 to 0.18 µm range), and the third at 0.07, with a band between 0.18 and 0.01 µm. The first two pore size populations, observed on the left side of Figure 2, correspond to the inter-particle filling between the granules. This result shows that the biochar supports possess a macroporosity network in addition to micropores. This can provide an accessible surface for the salt that can be homogeneously dispersed into the macropores. The possibility to charge into the support with a higher amount of salt allows for higher heat capacities. Moreover, if the salt can be efficiently dispersed on a larger, more accessible surface, the mass transfer is potentially ameliorated due to the thin layer of salt deposited. As a result, hydration kinetics can be improved. The other four composites were also characterized by Hg intrusion, and the results are shown in Figure 3. The pore size distribution of all four composites shows two main peaks located in the macropore range: 3 µm and 40 nm. A significant decrease in the 3 µm peak can be observed as the salt content increases. The decrease in the 3 µm peak also led to a reduction by 15% in terms of total porosity (from 70% to 55%) and more than 50% (from 1.89 to 0.81 cm^3^/g) in terms of pore volume.

**Figure 4:**
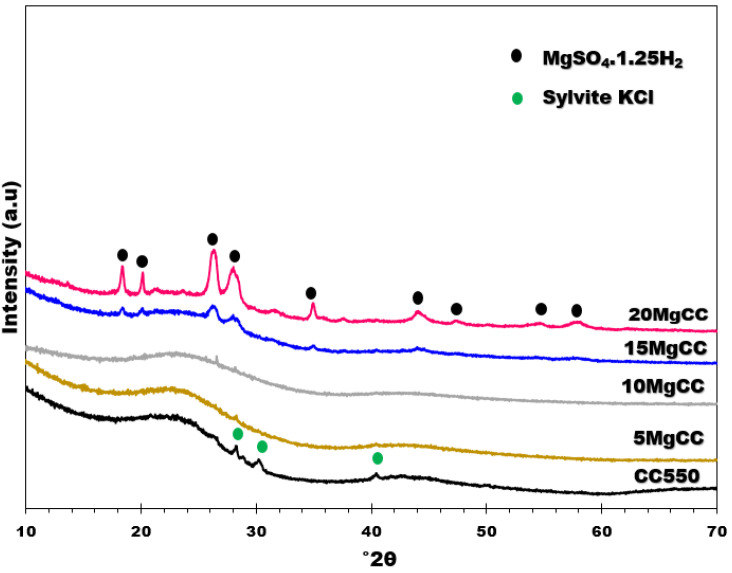
XRD patterns of CC550 and its composites.

XRD is a powerful method to investigate the eventual presence of crystalline impurities on the biochar and to identify the salt hydrate phases. The XRD patterns of all samples (Figure 4) displays an amorphous graphite peak at 23°2θ. This peak is less visible in the most charged sample (20MgCC). Three tiny sharp peaks can be identified at 28, 30, and 40°2θ in the XRD pattern of the biochar support, which were assigned to Sylvite KCl. Prakongkep et al. [39] investigated 14 biochars made from agricultural wastes such as corn cobs and found that K-minerals are contained in most of them, including Sylvite KCl. However, those peaks were no longer present in the XRD patterns of the composites. KCl was probably removed or overlapped during the MgSO_4_ impregnation procedure. According to the X’Pert HighScore Plus V4 software, the XRD patterns of the 15MgCC and 20MgCC samples showed sharp crystallinity diffraction peaks that were identified as MgSO_4_. 1.25H_2_O (Ref. Code: 00-028-0631), indicating that the dehydration at 150 °C was partial, as expected [40]. The intensity of the diffraction peaks indicates that more MgSO_4_. 1.25H_2_O crystallites were found in the 20MgCC sample. This can be explained by the fact that more salt was deposited on the surface of the biochar, resulting in clusters or thicker layers of MgSO_4_ salt that are more difficult to dehydrate. It should be noted that, to our knowledge, no literature has illuminated the formation of MgSO_4_. 1.25H_2_O (Ref. Code: 00-028-0631) on supported MgSO_4_. The confinement of the salt into the porous structure of biochar might be the reason for the stabilization of this phase. 

**Figure 5:**
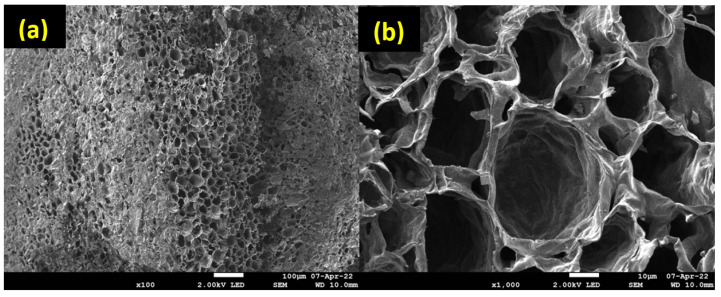
SEM images of CC550; (**a**) shows a scale of 100 µm, while (**b**) shows a scale of 10 µm.

**Figure 6:**
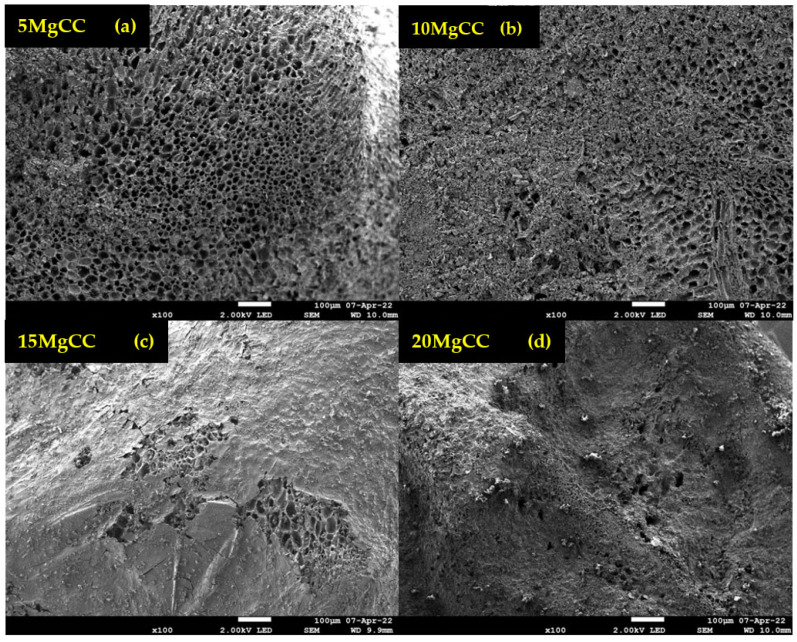
SEM images of the synthesized composites at different salt content. (**a**) 5MgCC; (**b**) 10MgCC; (**c**) 15MgCC; (**d**) 20MgCC.

SEM analyses were performed to examine the morphology and uniformity of salt deposition on the biochar surface. According to Figure 5, an irregular macropore network is observed, which confirms the results of Hg porosimetry. SEM analyses were also performed for the synthesized composites, and for further information, EDX mapping was conducted to analyze the salt distribution at the biochar surface. As shown in Figure 6, for the 5MgCC composite, the MgSO_4_ salt was deposited both on the external surface and inside the macropores. When the salt loading content started to increase, the macropore entrance became less and less accessible, as can be observed by the SEM picture in Figure 6 for the different composites with increasing salt content. EDX mapping showed, in each case, a homogeneous deposition of the salt on the external surface. Unfortunately, it is not possible to visualize the salt inside the pores; due to their depth, the electron beams cannot probe the internal surface (see Figures S1–S4 in the Supplementary Materials). 

### 2.2. Hydration Performances

**Figure 7:**
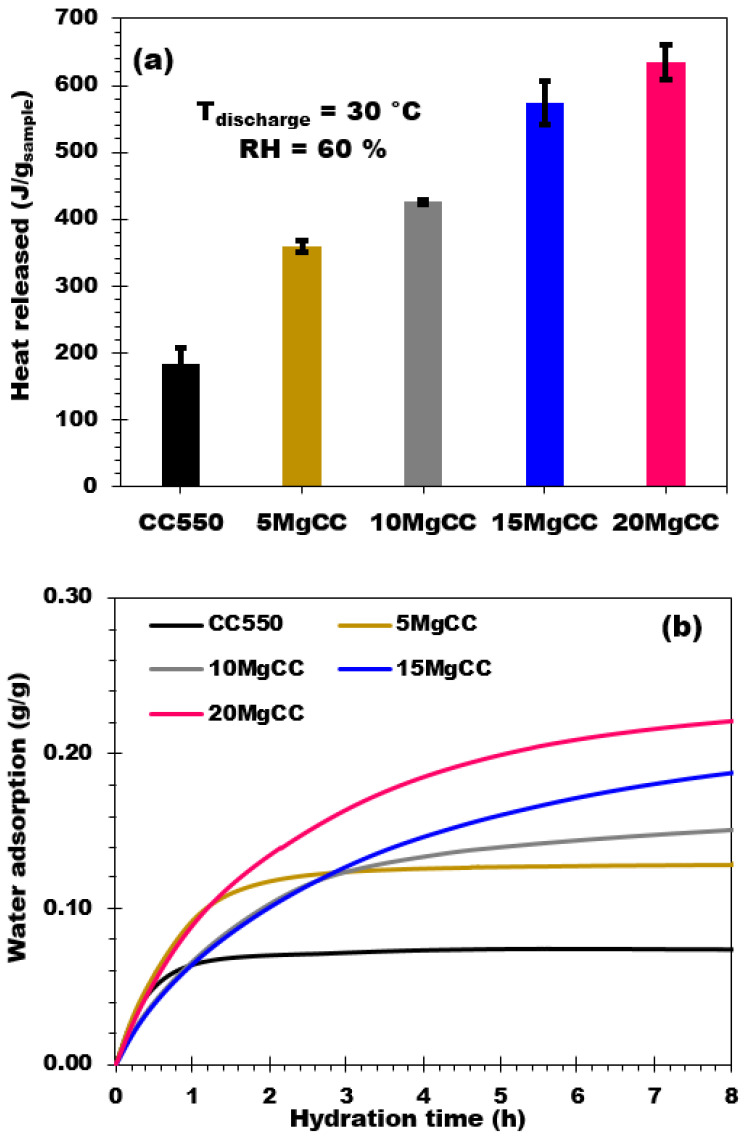
(**a**) Hydration behavior and (**b**) Water adsorption curves of biochar and synthesized adsorbent composites (RH = 60%; Tdischarge = 30 °C; 8 h of hydration).

The variation of the heat flow and the mass of the sample in relation to time were employed to determine the heat of hydration (Figure 7a) and the water adsorption capacity (labeled as “w_e_” in Equation (1)) (Figure 7b).

(1) $$w_{e}=\frac{m_{h}-m_{d}}{m_{d}}$$

where m_h_ (g) and m_d_ (g) represent the final masses of the hydrated and the dehydrated composite, respectively, and w_e_ is the water adsorption capacity (gH_2_O/g_sample_ or g/g in short).

The biochar support CC550 adsorbed 0.07 g of water per gram of dry composite, with a corresponding heat release of 183 J/g. The 5MgCC composite with 4.4% MgSO_4_ released 360 J/g of energy (water adsorption 0.126 g/g). As the salt concentration increased up to 19.5% (20MgCC composite), the heat production and the water uptake of the composites continued to increase to over 635 J/g and 0.235 g/g in water adsorption capacity for the 20MgCC composite. By the observation of the hydration curves, it can be observed that the hydration process is relatively fast and the isotherms quickly reach a plateau after 2 and 3 h for the support and the 5MgCC composite, respectively. Differently, the beginning of the hydration process presents a lower rate (lower slope of the isotherm) for the other composites; even after 8 h of hydration, the plateau (complete hydration) was not attained. 

**Figure 8:**
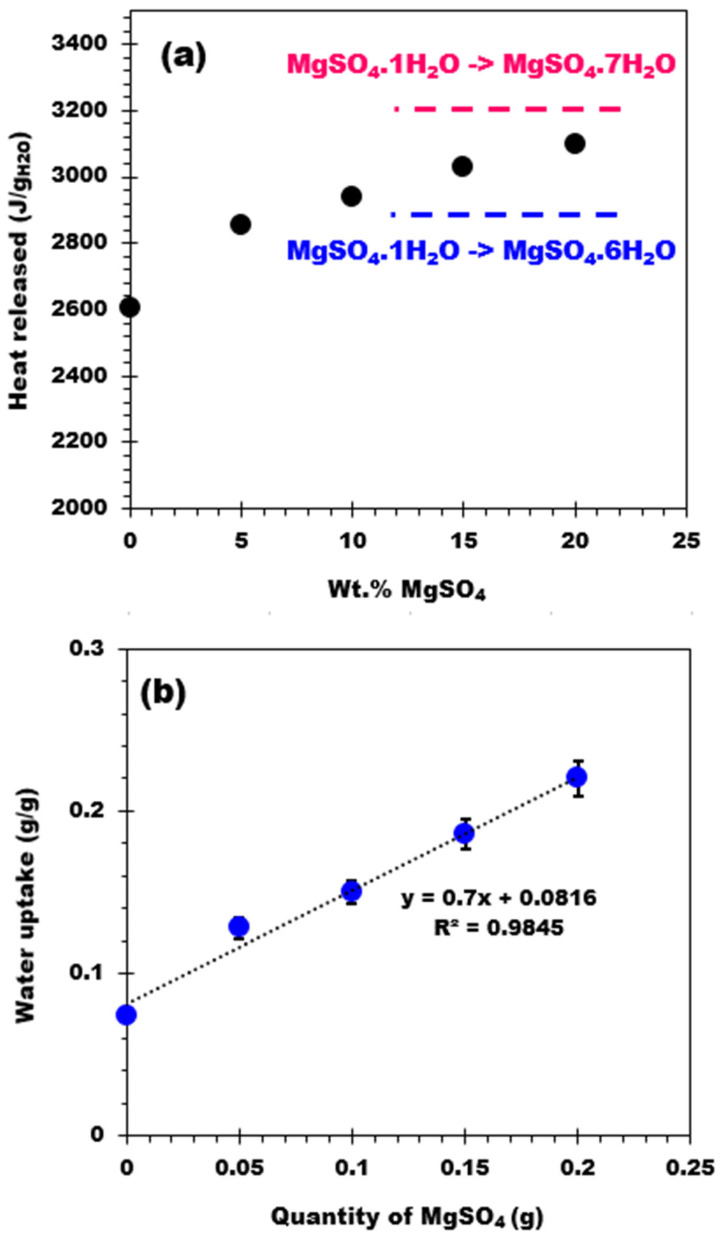
(**a**) Heat released (J/g water) as a function of the salt content and (**b**) Water uptake of the materials versus the salt content.

Table 2 reports the heat released by the biochar support and the four composites expressed as J/g_comp_ and J/g_water_. The heat released by the biochar is close to the water condensation heat (vapor → liquid) at 30 °C, which is approximately 2400 J/g_water_ [41]. This means that the heat released by the biochar is related to the condensation of water vapor on the biochar surface. The heat released (in J/g_water_) by the biochar and related composites is plotted as a function of the salt content in Figure 8a. The curve shows an increasing trend that tends to reach a value between 2900 and 3200 J/g_water_ [42] corresponding to the hydration of the salt from monohydrate to hexahydrate and from monohydrate to heptahydrate, respectively. From this observation, one can conclude that after the hydration process, the final hydration state is between six and seven molecules of water. Similar to the heat release behavior, the water adsorption has also been plotted as a function of the salt content, showing a linear increasing trend (Figure 8b). These results prove that all the composites adsorb water vapor similarly, regardless of the amount of salt deposited on the biochar support (no apparent mass transfer limitation).

Table 3 compares the performance of the 20MgCC sample with other composite storage materials (also impregnated with MgSO_4_) reported in the literature. Supporting MgSO_4_ on the corncob biochars seems to give origin to composites with a higher heat storage capacity than other composites previously reported (even when containing higher quantities of salt).

### 2.3. Hydration Kinetic Modeling

**Figure 9:**
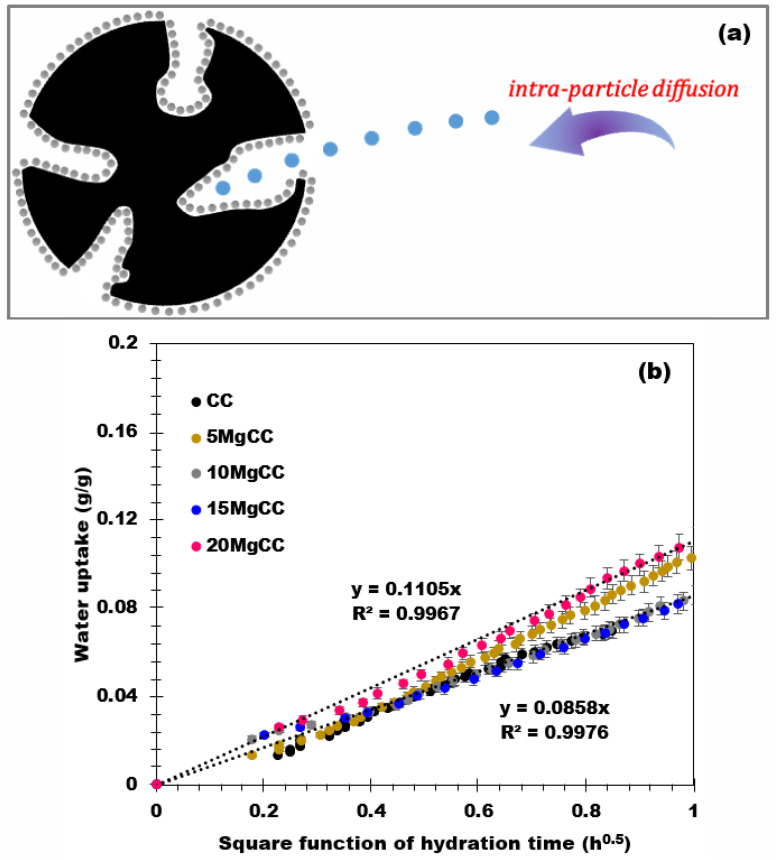
(**a**) Representation of the intra-particle diffusion model and (**b**) Water uptake versus the square root of the hydration time.

Various kinetic models are available in the literature and have been applied for the study of the kinetics of hydration of sorbent materials [48]. Among the models, the intra-particle diffusion model (Figure 9a), described by Equation (2) below, achieves good numerical approximations when applied to the hydration of biochar and related composites and better describes the physical phenomena involved during the water uptake.

(2) $$q_{t}=k_{i}*t^{0.5}+C$$

with *k_i_* as the intra-particle diffusion rate constant (g/g^1^/h^0.5^), *t* the hydration time (h), and *C* represents the material transfer resistance (g/g).

This model assumes the diffusion of water on composites with a homogeneous dispersion of the salt. As shown in Figure 9b, the amount of water adsorbed is a linear function of the square root of the hydration time for all the materials. The intra-particle diffusion rate constant of each material was then deduced and showed a similar outcome between 0.09 and 0.11 for all samples. These results validate the model assumptions and confirm that the intra-particle model provides a good description of the hydration phenomena for these types of materials.

### 2.4. Cyclability Investigation

**Figure 10:**
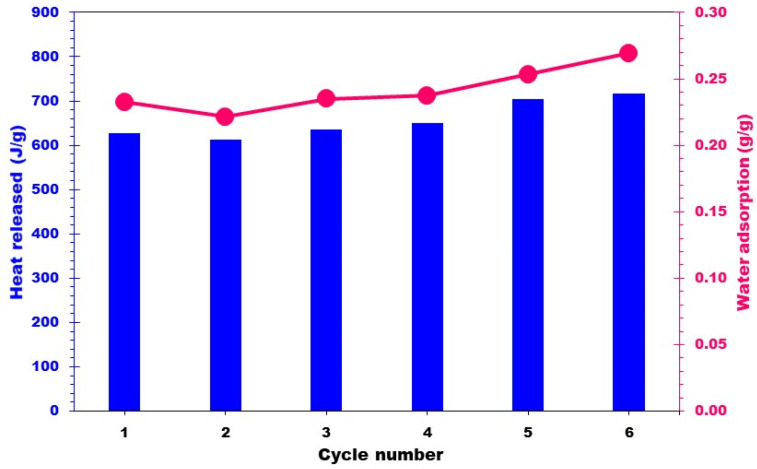
Heat released from the composite 20MgCC for six successive cycles of dehydration/hydration.

**Figure 11:**
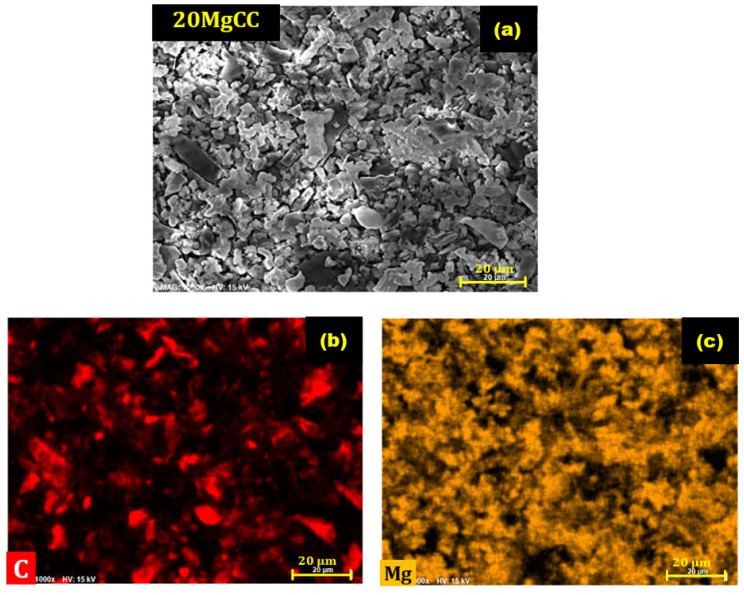
SEM/EDX images of the composite 20MgCC after six cycles. (**a**) SEM image of 20MgCC; (**b**) EDX mapping of carbon (C); (**c**) EDX mapping of Magnesium (Mg).

To test the cyclability of the best performing composite, the 20MgCC sample was submitted to six consecutive cycles of hydration (30 °C and an RH of 60%) and dehydration (150 °C). To determine whether the hydration behavior was consistent, the heat generated following each cycle was recorded and compared to the earlier ones. Figure 10 shows a negligible variation in the heat released and water uptake, confirming the good cyclability of the composite. Figure 11 shows the EDX images after six cycles of 20MgCC. The salt remained well dispersed on the support, confirming the stability of the synthesized 20MgCC composite.

## 3. Materials and Methods

### 3.1. Preparation Method of Composites

The biochar was prepared in a pilot pyrolyzer at the RAPSODEE-UMR CNRS 5302 laboratory, following the procedure detailed in [19]. In the present case, the parent biomass was composed of corncobs collected in the Alsace region (to answer circular economy issues) and dried at 105 °C for 24 h. They were then pyrolyzed at 550 °C under an N_2_ flow rate of 100 mL/min. The pyrolysis temperature was reached with a heating rate of 4 °C/min and maintained for 1.5 h. The biochar obtained after the pyrolysis was sieved between 2.5–4 mm and labeled CC550. The obtained biochar was used as a porous support for MgSO_4_ salt hydrate.

The impregnation method was used to deposit MgSO_4_ (MgSO_4_. 7H_2_O, 99.9% from Sigma-Aldrich (Saint Quentin Fallavier, France) onto the CC550 porous support. The method consists of intimately mixing the porous support with a MgSO_4_ solution. Prior to impregnation, to eliminate water from the pores, the biochar was dried in an oven at 150 °C. The aqueous solution of MgSO_4_ was then added over the dried support. The mixture was subjected to constant mixing for 4 h to homogenize the impregnation. The obtained powders were then dried at 60 °C for 12 h and successively at 150 °C for 12 h more. Four composite adsorbents were synthesized and then designated as xMgCC (Table 1), with x is the theoretical content of MgSO_4_ in the composites. The real salt content was then verified by X-ray fluorescence.

### 3.2. Techniques of Characterizations

The parameters and conditions used to characterize the composites by X-Ray Diffraction (XRD) analyses, X-Ray Fluorescence, CO_2_ adsorption, and Scanning Electron Microscope (SEM) are detailed in our previously published papers [9,43,49].

Mercury porosimetry analysis on the biochar was subcontracted to a FiLAB provider that performed the analysis with an Autopore IV device from Micrometrics. The sample was degassed at ambient temperature and 50 µmHg pressure for 3 h. Mercury intrusion was performed at 22 °C with a contact angle of 130 °C and a pressure range of 0.52 to 60,000 psia (0.036 to 4137 bar), covering a pore range of 350 µm to 3 nm. 

### 3.3. Heat Released and Water Adsorption Experiments

The apparatus and procedure employed to examine the performance of composites are reported in our previously published papers [9,43]. The samples (15–25 mg) were first dehydrated at 150 °C under a flow of dry air (30 mL/min) followed by 3 h in a 150 °C isotherm to perform the dehydration. The dehydration temperature was selected on the basis of the temperature that can be obtained in a real application by using flat-plate solar heat collectors [47,50]. After the dehydration step, the sample was cooled to 30 °C. The sample was kept at this temperature in dry air until the DSC signal attained a stable baseline. Then the relative humidity (RH) of the air flow was augmented to 60% to begin the hydration phase. To reach a complete rehydration, the samples were hydrated for 8 h until the DSC signal went back to the baseline. The TG–DSC apparatus used in this study has inherent experimental errors, with an accuracy of ±0.01 °C for temperature measurements and ±10^−6^ g for mass measurements. To ensure an accurate calculation of the dehydration/hydration heat, a standardized procedure was followed. The dehydration/hydration process was conducted after stabilizing the DSC and TGA signals. This procedure was carried out for both the empty crucible (blank experiment) and each sample. By subtracting the signals from the blank experiment, the dehydration/hydration heat (expressed in J/g_sample_) was obtained. The estimation of the hydration heat values includes an error of approximately 15 kJ/kg_sample_, which takes into account systematic errors in heat flow acquisition, mass measurement obtained by the coupled balance, and the integration of the calorimetric peaks. To ensure the reliability and consistency of the results, all experiments were performed in duplicate. The obtained results from these duplicate experiments exhibited close agreement, indicating no significant modifications in heat and mass transfer. This enhances the reproducibility and reliability of our findings.

## 4. Conclusions

In conclusion, this work presents the synthesis and characterization of a novel series of composites based on corncob, an agricultural waste, for long-term heat storage applications. The composites were successfully synthesized by impregnating MgSO_4_ onto corncob biochar and extensively characterized using XRD, BET, and SEM techniques. The CO_2_ sorption results revealed that the corncob biochar support exhibited a small microporous surface area of 175 m^2^/g while displaying an enhanced macroporous structure. This unique feature allows for the homogeneous deposition of a large quantity of salt within the composites. Among the prepared series, the 20MgCC composite demonstrated the best performance, with an impressive energy storage density of 635 J/g at Tads = 30 °C and RH = 60%. This achievement highlights the superior energy storage capabilities of corncob-based composites developed in this work compared to the existing literature (please see Table 3). The hydration mechanism of the synthesized composites was well described by the intra-particle model, wherein water molecules diffuse through a homogeneous layer of salt deposited on the surface and inside the larger pores of the composites. The cyclability of the MgSO_4_/biochar composites was confirmed through six hydration/dehydration cycles, demonstrating the stability and durability of the 20MgCC composite. One of the significant achievements of this work is the utilization of biosourced materials, specifically corncob-derived biochar, as a constituent of heat storage composites. This accomplishment contributes to advancing green management practices in thermal energy applications. The use of biomass sources for biochar production offers opportunities for circular economy practices, reduced carbon footprint, and enhanced system sustainability. The abundance of biomass types available for biochar production opens up new avenues for the research and development of environmentally friendly heat storage materials. Overall, this study provides valuable insights into the synthesis and characterization of corncob-based composites for long-term heat storage, showcasing their potential for sustainable energy management. The superior performance of the 20MgCC composite and the utilization of biosourced materials demonstrate the possibility of achieving more efficient and environmentally friendly heat storage solutions. Further research and comparative studies with a broader range of composites will be valuable for a comprehensive evaluation of the specific achievements and advantages of the developed materials over the existing literature.

## Figures and Tables

**Table 1:** Amount of salt determined by XRF and hydration experiments results.

Sample	MgSO_4_ Content (wt%)	Heat Released (J/g_sample_)	Water Adsorption (g/g)
CC550	0.0	183	0.07
5MgCC	4.42	360	0.13
10MgCC	9.10	426	0.16
15MgCC	12.60	574	0.21
20MgCC	19.51	635	0.24

**Table 2:** Heat released during the hydration of the biochar and its composites.

Sample	Heat Released (J/g_comp_)	Heat Released (J/g_H2O_)
CC	183	2607
5MgCC	360	2854
10MgCC	426	2940
15MgCC	574	3031
20MgCC	635	3100

**Table 3:** Comparison of 20MgCC with other composites based on sulfate in the literature.

Composite Adsorbents	Setting Conditions	Energy Storage Density (J/g)	Ref.
20MgCC	T_hyd_ = 30 °C; RH = 60%	635	This study
20-MgSO_4_/HAP	T_hyd_ = 30 °C; RH = 60%	464	[43]
60-MgSO_4_/Diatomite (D60)	T_hyd_ = 25 °C; RH = 80%	773	[44]
50-MgSO_4_/Expanded graphite (EG50)	T_hyd_ = 25 °C; RH = 85%	496.4	[45]
MgSO_4_/13x with %MgSO_4_ up to 20%	T_hyd_ = 25 °C; RH = 60%	510–575	[46]
MgSO_4_/zeolite Modernite	T_hyd_ = 22 °C; RH = 56%	507	[47]

## Data Availability

The original data related to this research can be obtained at any time via the corresponding author’s email: simona.bennici@uha.fr.

## Supplementary Materials

The following supporting information can be downloaded at: https://www.mdpi.com/article/10.3390/molecules28145381/s1, Figure S1: SEM images and EDX mappings of 5MgCC. Figure S2: SEM images and EDX mappings of 10MgCC. Figure S3: SEM images and EDX mappings of 15MgCC. Figure S4: SEM images and EDX mappings of 20MgCC.

## References

[1] Cabeza L.F., de Gracia A., Zsembinszki G., Borri E. (2021). Perspectives on thermal energy storage research. Energy.

[2] Carrillo A.J., González-Aguilar J., Romero M., Coronado J.M. (2019). Solar Energy on Demand: A Review on High Temperature Thermochemical Heat Storage Systems and Materials. Chem. Rev..

[3] Lin J., Zhao Q., Huang H., Mao H., Liu Y., Xiao Y. (2021). Applications of low-temperature thermochemical energy storage systems for salt hydrates based on material classification: A review. Sol. Energy.

[4] Liu H., Wang W., Zhang Y. (2021). Performance gap between thermochemical energy storage systems based on salt hydrates and materials. J. Clean. Prod..

[5] Xu J., Li T., Yan T., Chao J., Wang R. (2021). Dehydration kinetics and thermodynamics of magnesium chloride hexahydrate for thermal energy storage. Sol. Energy Mater. Sol. Cells.

[6] N’Tsoukpoe K.E., Schmidt T., Rammelberg H.U., Watts B.A., Ruck W.K.L. (2014). A systematic multi-step screening of numerous salt hydrates for low temperature thermochemical energy storage. Appl. Energy.

[7] Zhao Q., Lin J., Huang H., Xie Z., Xiao Y. (2022). Enhancement of heat and mass transfer of potassium carbonate-based thermochemical materials for thermal energy storage. J. Energy Storage.

[8] Zbair M., Bennici S. (2021). Survey Summary on Salts Hydrates and Composites Used in Thermochemical Sorption Heat Storage: A Review. Energies.

[9] Bennici S., Dutournié P., Cathalan J., Zbair M., Nguyen M.H., Scuiller E., Vaulot C. (2022). Heat storage: Hydration investigation of MgSO_4_/active carbon composites, from material development to domestic applications scenarios. Renew. Sustain. Energy Rev..

[10] Linnow K., Niermann M., Bonatz D., Posern K., Steiger M. (2014). Experimental Studies of the Mechanism and Kinetics of Hydration Reactions. Energy Procedia.

[11] Liu W.-J., Jiang H., Yu H.-Q. (2019). Emerging applications of biochar-based materials for energy storage and conversion. Energy Environ. Sci..

[12] Saning A., Herou S., Dechtrirat D., Ieosakulrat C., Pakawatpanurut P., Kaowphong S., Thanachayanont C., Titirici M.-M., Chuenchom L. (2019). Green and sustainable zero-waste conversion of water hyacinth (Eichhornia crassipes) into superior magnetic carbon composite adsorbents and supercapacitor electrodes. RSC Adv..

[13] Senthil C., Lee C.W. (2021). Biomass-derived biochar materials as sustainable energy sources for electrochemical energy storage devices. Renew. Sustain. Energy Rev..

[14] Tursi A. (2019). A review on biomass: Importance, chemistry, classification, and conversion. Biofuel Res. J..

[15] Gunasekaran S.S., Elumalali S.K., Kumaresan T.K., Meganathan R., Ashok A., Pawar V., Vediappan K., Ramasamy G., Karazhanov S.Z., Raman K. (2018). Partially graphitic nanoporous activated carbon prepared from biomass for supercapacitor application. Mater. Lett..

[16] Ma Y., Yao D., Liang H., Yin J., Xia Y., Zuo K., Zeng Y.-P. (2020). Ultra-thick wood biochar monoliths with hierarchically porous structure from cotton rose for electrochemical capacitor electrodes. Electrochim. Acta.

[17] Jaswal R., Shende A., Nan W., Amar V., Shende R. (2019). Hydrothermal Liquefaction and Photocatalytic Reforming of Pinewood (Pinus ponderosa)-Derived Acid Hydrolysis Residue for Hydrogen and Bio-oil Production. Energy Fuels.

[18] Hou J., Jiang K., Tahir M., Wu X., Idrees F., Shen M., Cao C. (2017). Tunable porous structure of carbon nanosheets derived from puffed rice for high energy density supercapacitors. J. Power Sources.

[19] Frikha K., Limousy L., Arif M.B., Thevenin N., Ruidavets L., Zbair M., Bennici S. (2021). Exhausted Grape Marc Derived Biochars: Effect of Pyrolysis Temperature on the Yield and Quality of Biochar for Soil Amendment. Sustainability.

[20] Giudicianni P., Cardone G., Ragucci R. (2013). Cellulose, hemicellulose and lignin slow steam pyrolysis: Thermal decomposition of biomass components mixtures. J. Anal. Appl. Pyrolysis.

[21] Freddo A., Cai C., Reid B.J. (2012). Environmental contextualisation of potential toxic elements and polycyclic aromatic hydrocarbons in biochar. Environ. Pollut..

[22] Bruckman V.J., Varol E.A., Liu J., Uzun B.B. (2016). Biochar.

[23] Tan X., Liu S., Liu Y., Gu Y., Zeng G., Hu X., Wang X., Liu S., Jiang L. (2017). Biochar as potential sustainable precursors for activated carbon production: Multiple applications in environmental protection and energy storage. Bioresour. Technol..

[24] El-Naggar A., Lee S.S., Rinklebe J., Farooq M., Song H., Sarmah A.K., Zimmerman A.R., Ahmad M., Shaheen S.M., Ok Y.S. (2019). Biochar application to low fertility soils: A review of current status, and future prospects. Geoderma.

[25] Nanda S., Dalai A.K., Berruti F., Kozinski J.A. (2016). Biochar as an Exceptional Bioresource for Energy, Agronomy, Carbon Sequestration, Activated Carbon and Specialty Materials. Waste Biomass Valorization.

[26] Ahmad M., Rajapaksha A.U., Lim J.E., Zhang M., Bolan N., Mohan D., Vithanage M., Lee S.S., Ok Y.S. (2014). Biochar as a sorbent for contaminant management in soil and water: A review. Chemosphere.

[27] Zheng T., Ouyang S., Zhou Q. (2023). Synthesis, characterization, safety design, and application of NPs@BC for contaminated soil remediation and sustainable agriculture. Biochar.

[28] Fang Y., Singh B., Singh B.P. (2015). Effect of temperature on biochar priming effects and its stability in soils. Soil Biol. Biochem..

[29] Suárez-Abelenda M., Kaal J., McBeath A.V. (2017). Translating analytical pyrolysis fingerprints to Thermal Stability Indices (TSI) to improve biochar characterization by pyrolysis-GC-MS. Biomass Bioenergy.

[30] Ulusal A., Apaydın Varol E., Bruckman V.J., Uzun B.B. (2021). Opportunity for sustainable biomass valorization to produce biochar for improving soil characteristics. Biomass Convers. Biorefin..

[31] Kambo H.S., Dutta A. (2015). A comparative review of biochar and hydrochar in terms of production, physico-chemical properties and applications. Renew. Sustain. Energy Rev..

[32] Tkemaladze G.S., Makhashvili K.A. (2016). Climate changes and photosynthesis. Ann. Agrar. Sci..

[33] Kaltschmitt M. (2013). Renewable Energy Renewable Energy from Biomass renewable energy from Biomass, Introduction. Renewable Energy Systems.

[34] Zhang Y., He M., Wang L., Yan J., Ma B., Zhu X., Ok Y.S., Mechtcherine V., Tsang D.C.W. (2022). Biochar as construction materials for achieving carbon neutrality. Biochar.

[35] Chen Y., Guo X., Liu A., Zhu H., Ma T. (2021). Recent progress in biomass-derived carbon materials used for secondary batteries. Sustain. Energy Fuels.

[36] Deng J., Li M., Wang Y. (2016). Biomass-derived carbon: Synthesis and applications in energy storage and conversion. Green Chem..

[37] Sheldon R.A. (2014). Green and sustainable manufacture of chemicals from biomass: State of the art. Green Chem..

[38] Ma L.-L., Hu X., Liu W.-J., Li H.-C., Lam P.K.S., Zeng R.J., Yu H.-Q. (2021). Constructing N, P-dually doped biochar materials from biomass wastes for high-performance bifunctional oxygen electrocatalysts. Chemosphere.

[39] Prakongkep N., Gilkes R.J., Wiriyakitnateekul W. (2015). Forms and solubility of plant nutrient elements in tropical plant waste biochars. J. Plant Nutr. Soil Sci..

[40] van Essen V.M., Zondag H.A., Gores J.C., Bleijendaal L.P.J., Bakker M., Schuitema R., van Helden W.G.J., He Z., Rindt C.C.M. (2009). Characterization of MgSO_4_ Hydrate for Thermochemical Seasonal Heat Storage. J. Sol. Energy Eng..

[41] Ayou D.S., Currás M.R., Salavera D., García J., Bruno J.C., Coronas A. (2014). Performance analysis of absorption heat transformer cycles using ionic liquids based on imidazolium cation as absorbents with 2,2,2-trifluoroethanol as refrigerant. Energy Convers. Manag..

[42] Grevel K.D., Majzlan J., Benisek A., Dachs E., Steiger M., Fortes A.D., Marler B. (2012). Experimentally determined standard thermodynamic properties of synthetic MgSO_4_·4H_2_O (Starkeyite) and MgSO_4_·3H_2_O: A revised internally consistent thermodynamic data set for magnesium sulfate hydrates. Astrobiology.

[43] Nguyen M.H., Zbair M., Dutournié P., Gervasini A., Vaulot C., Bennici S. (2022). Toward new low-temperature thermochemical heat storage materials: Investigation of hydration/dehydration behaviors of MgSO_4_/Hydroxyapatite composite. Sol. Energy Mater. Sol. Cells.

[44] Zhang Y., Miao Q., Jia X., Jin Y., Li Z., Tan L., Ding Y. (2021). Diatomite-based magnesium sulfate composites for thermochemical energy storage: Preparation and performance investigation. Sol. Energy.

[45] Miao Q., Zhang Y., Jia X., Tan L., Ding Y. (2021). MgSO_4_-expanded graphite composites for mass and heat transfer enhancement of thermochemical energy storage. Sol. Energy.

[46] Wang Q., Xie Y., Ding B., Yu G., Ye F., Xu C. (2019). Structure and hydration state characterizations of MgSO_4_-zeolite 13x composite materials for long-term thermochemical heat storage. Sol. Energy Mater. Sol. Cells.

[47] Whiting G., Grondin D., Bennici S., Auroux A. (2013). Heats of water sorption studies on zeolite–MgSO_4_ composites as potential thermochemical heat storage materials. Sol. Energy Mater. Sol. Cells.

[48] Wang J., Guo X. (2020). Adsorption kinetic models: Physical meanings, applications, and solving methods. J. Hazard. Mater..

[49] Nguyen M.H., Zbair M., Dutournié P., Bennici S. (2023). Thermochemical sorption heat storage: Investigate the heat released from activated carbon beads used as porous host matrix for MgSO_4_ salt. J. Energy Storage.

[50] Donkers P.A.J., Sögütoglu L.C., Huinink H.P., Fischer H.R., Adan O.C.G. (2017). A review of salt hydrates for seasonal heat storage in domestic applications. Appl. Energy.

