# Supplementary material for: Corn Cobs’ Biochar as Green Host of Salt Hydrates for Enhancing the Water Sorption Kinetics in Thermochemical Heat Storage Systems
Source: Molecules. 2023 Jul 13;28(14):5381. doi: 10.3390/molecules28145381 (PMC10383902; doi:10.3390/molecules28145381)
Supplement: Supplementary file 1 [file molecules-28-05381-s001.zip › molecules-2470008-supplementary.pdf]

Supplementary Materials

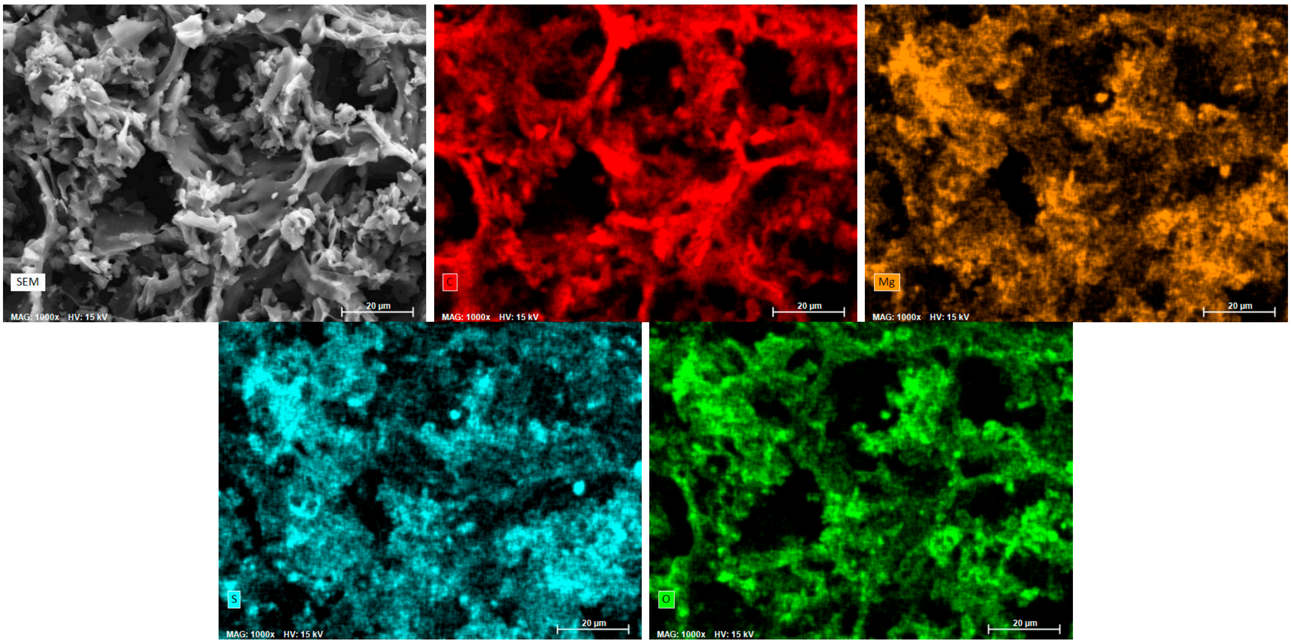

Figure S1: SEM images and EDX mappings of 5MgCC.

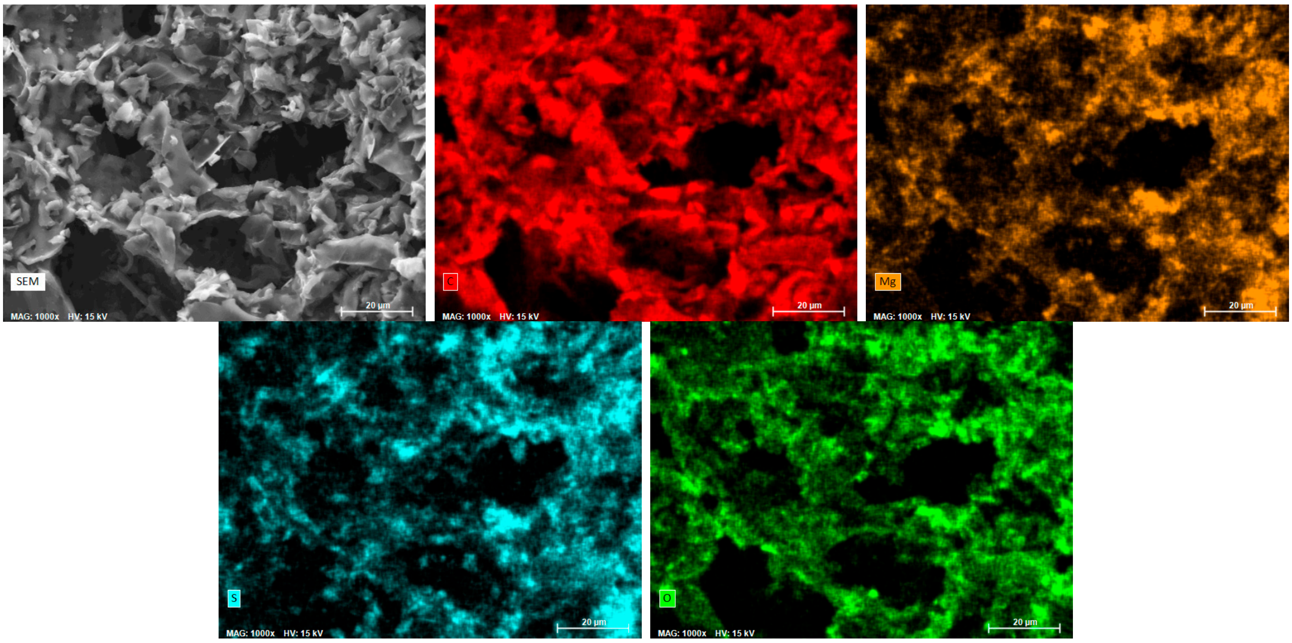

Figure S2: SEM images and EDX mappings of 10MgCC.

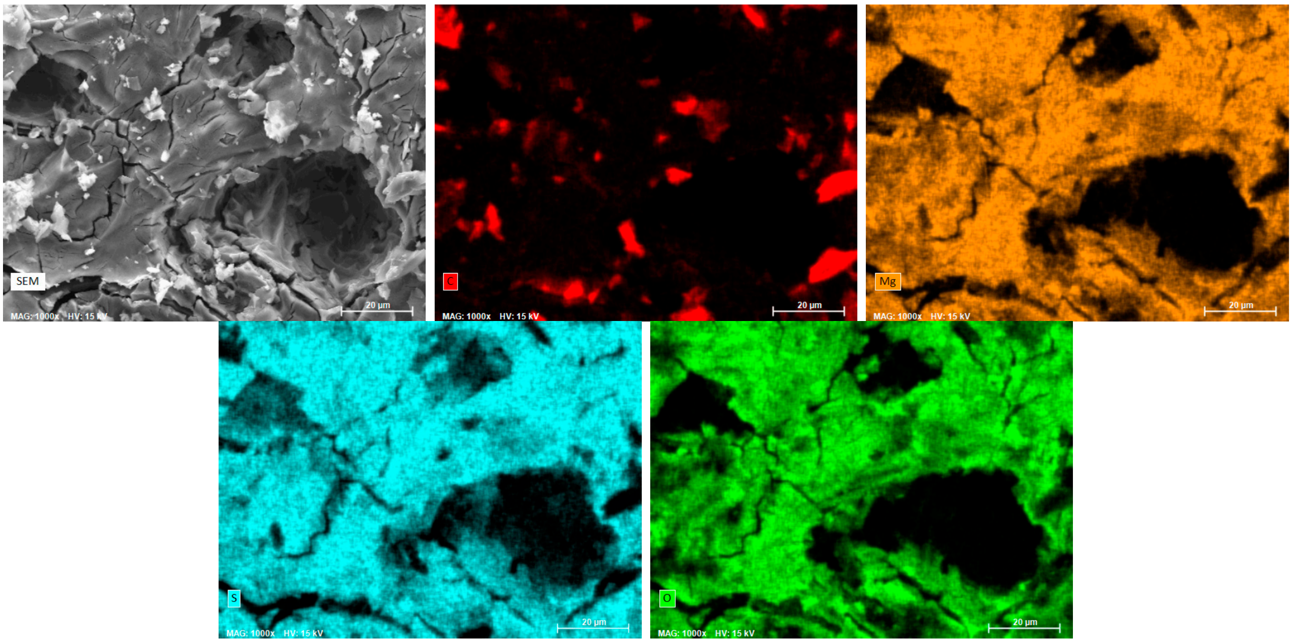

Figure S3: SEM images and EDX mappings of 15MgCC.

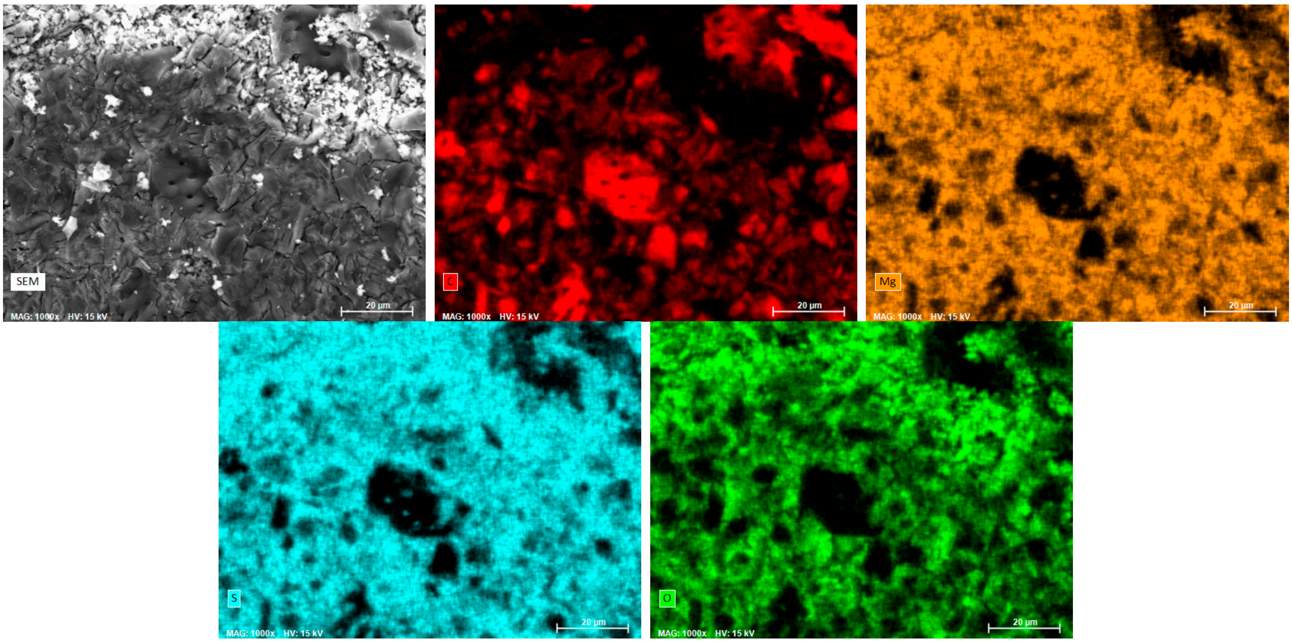

Figure S4: SEM images and EDX mappings of 20MgCC.
